# Recent advances in access to overcome cancer drug resistance by nanocarrier drug delivery system

**DOI:** 10.20517/cdr.2023.16

**Published:** 2023-06-20

**Authors:** Xiangyu Sun, Ping Zhao, Jierou Lin, Kun Chen, Jianliang Shen

**Affiliations:** ^1^Medicines and Equipment Department, Beijing Chaoyang Emergency Medical Rescuing Center, Chaoyang District, Beijing 100026, China.; ^2^School of Chemistry and Chemical Engineering, Guangdong Pharmaceutical University, Education Meg Centre, Guangzhou 510006, Guangdong, China.; ^3^Beijing Chaoyang Emergency Medical Rescuing Center, Chaoyang District, Beijing 100026, China.; ^4^School of Ophthalmology & Optometry, School of Biomedical Engineering, Wenzhou Medical University, Wenzhou 325035, Zhejiang, China.; ^5^Wenzhou Institute, University of Chinese Academy of Sciences, Wenzhou 325001, Zhejiang, China.

**Keywords:** Cancer, nanomedicine, nanomaterials, drug delivery, multidrug resistance

## Abstract

Cancer is currently one of the most intractable diseases causing human death. Although the prognosis of tumor patients has been improved to a certain extent through various modern treatment methods, multidrug resistance (MDR) of tumor cells is still a major problem leading to clinical treatment failure. Chemotherapy resistance refers to the resistance of tumor cells and/or tissues to a drug, usually inherent or developed during treatment. Therefore, an urgent need to research the ideal drug delivery system to overcome the shortcoming of traditional chemotherapy. The rapid development of nanotechnology has brought us new enlightenments to solve this problem. The novel nanocarrier provides a considerably effective treatment to overcome the limitations of chemotherapy or other drugs resulting from systemic side effects such as resistance, high toxicity, lack of targeting, and off-target. Herein, we introduce several tumor MDR mechanisms and discuss novel nanoparticle technology applied to surmount cancer drug resistance. Nanomaterials contain liposomes, polymer conjugates, micelles, dendrimers, carbon-based, metal nanoparticles, and nucleotides which can be used to deliver chemotherapeutic drugs, photosensitizers, and small interfering RNA (siRNA). This review aims to elucidate the advantages of nanomedicine in overcoming cancer drug resistance and discuss the latest developments.

## INTRODUCTION

Physiologically, the formation of tumor cells is an uncontrolled and unnecessary growth of cells that require a large number of nutrients. According to the world health organization (WHO) report, cancer is the first or second leading cause of death in 112 out of 183 countries. One of the most common causes of death in cancer patients is the development of multidrug resistance (MDR)^[1,2]^. The tendency of tumor cell drug resistance and its mortality rate is increasing yearly^[3]^. Based on the development of the current medical level, chemotherapy is a conventional treatment for tumors. Although chemotherapy has made a great success, due to the lack of targeting and limited bioavailability, its efficacy is still facing severe challenges in clinical practice. More importantly, tumor cells will generate MDR, which can enhance the response threshold of other cytotoxic drugs after long-term administration of chemotherapy drugs^[4,5]^. Novel molecular targeted therapy and immunotherapy have become methods to overcome the lack of specificity of traditional chemotherapy drugs^[6]^. However, due to the development of drug resistance, cancer cells still can evade the cytotoxicity of newer molecular-targeted therapeutic drugs. Therefore, resistance to drugs is a common cause of death in cancer patients treated with conventional chemotherapy or novel targeted drugs. MDR is a highly heterogeneous disease state. When a tumor cell becomes resistant to a single anticancer drug, it may become cross-resistant to a range of drugs with different structures or mechanisms. It is one of the key factors for recurrence and metastasis of tumor patients^[7]^.

The decrease of drug accumulation in cells is the common cause of drug resistance in tumor cells. At present, it is generally believed that tumor cells develop tolerance to drug toxicity in two ways: one is to prevent drugs from reaching the target site through a series of self-regulation when drugs enter the cell from the outside; the other is to pump drugs into the cytoplasm directly outside the cell so that the concentration of drugs available inside the cell is reduced. Up to now, the mechanism of tumor drug resistance has not been fully understood^[8,9]^. Cancer resistance is divided into two broad categories: primary resistance, with an early tumor progression, without prior tumor response, and secondary (acquired) resistance, which occurs after initial tumor responses^[10,11]^. Among them, primary resistance may be mainly related to the lack of target dependence or the presence of other targets. The main mechanisms of acquired resistance include on-target resistance alterations, bypass alterations in the same pathway or connecting pathways, and changes in phenotypic transformation of tumor cells^[12,13]^.

The application of nanomaterials in the medical field is also called nanomedicine. This new treatment method can combine the delivery of drugs and improve the treatment effect while protecting normal tissues from side effects. In this paper, we start with the mechanism of tumor drug resistance and introduce the molecular mechanisms related to tumor drug resistance, such as the ATP-binding cassette (ABC) transporter family and MDR mediated by the enzyme system. Next, the research of a nano-drug delivery system in reversing tumor drug resistance was introduced, including the value of nanocarrier platforms in improving the solubility of chemotherapy drugs, improving drug targeting, multidrug delivery, and others. Finally, this paper expounds on the nano-drug delivery system reversal of tumor resistance to remedy the technical advantages and application prospects.

## MECHANISMS OF RESISTANCE IN CANCER

**Figure 1:**
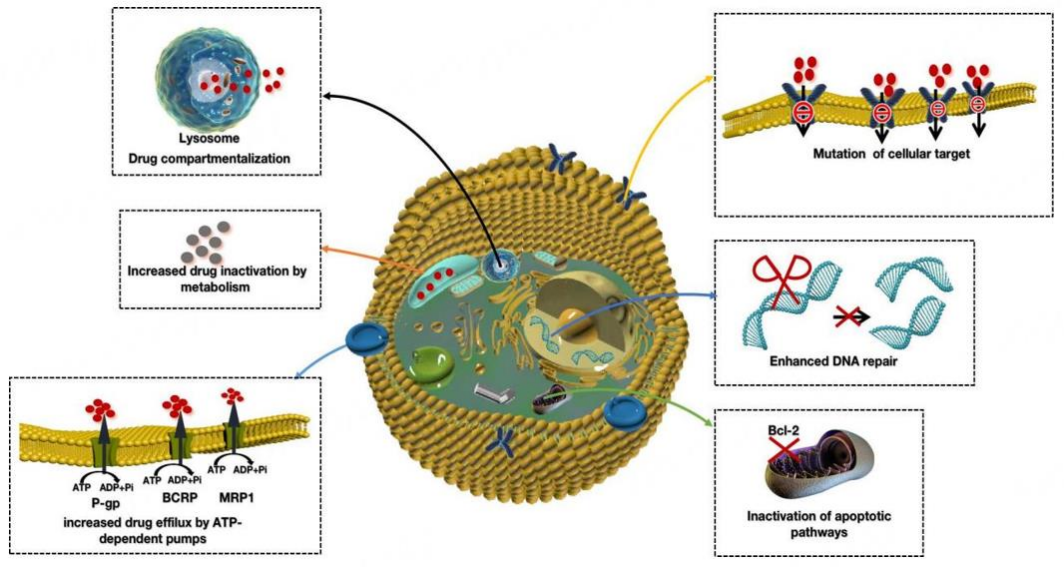
This diagram depicts the expulsion of cells by efflux transporters, including P-gp, BCRP, and MRP-1. Other mechanization includes drug inactivation by increasing the expression of enzymes that metabolize drugs and the development of resistance. Cells can develop resistance by changing the target of a drug, thus disabling the drug. Drug isolation into lysosomes can cause drug inactivation and cause cancer cells to acquire resistance. Drugs inhibit cell apoptosis and produce drug resistance by interfering with the genetic cycle of cells. BCRP: Breast cancer resistance protein; Bcl-2: B-cell leukemia protein 2; MRP: multidrug resistance-related protein; P-gp: p-glycoprotein.

More than half of cancer deaths are due to the generation of drug resistance^[2]^. The drug resistance of tumor cells is involved in a variety of mechanisms, including the increase of MDR during long-term chemotherapy, DNA repair ability enhancement, genetic mutation, heterogeneous biological metabolism, blocked apoptosis pathway, microenvironment changes, and drug target alteration, among others^[14-16]^. Any one or more of the above mechanisms will reduce the drug efficacy and increase the difficulty of tumor treatment. Therefore, from the perspective of molecular mechanisms, the study of molecular markers and molecular targets for reversing tumor resistance is the focus of tumor therapy [Figure 1]. Common MDR mechanisms that lead to drug resistance in tumor cells are briefly highlighted in the following sections.

### Efflux of drugs mediated by ABC transporter family

The ABC transporter is a transmembrane protein that relies on the energy produced by the hydrolysis of ATP to shuttle the substrate through the channels of the cell membrane^[17,18]^. It is mainly responsible for regulating the distribution, absorption, and excretion of various compounds. Among the ABC transporters that have been discovered, the primary proteins related to tumor cell drug resistance are p-glycoprotein (P-gp/MDR-1, or ABCB1)^[19]^, multidrug resistance-related protein (MRP-1/ABCC1)^[20]^, breast cancer resistance protein (BCRP/ABCG2)^[21]^. In follow-up studies, scientists found that these proteins were up-regulated in various MDR cancer cells^[22-24]^. Among them, the most typical cause of MDR is the overexpression of P-gp. P-gp has a self-protection mechanism, which can efflux most chemotherapeutic drugs out of the cell to reduce intracellular drug accumulation. The substrates of P-gp protein are widespread, including varieties of antitumor drugs, such as paclitaxel, cyclophosphamide, DOX, and emerging molecular targeted drugs^[25-28]^. In brief, resistance is more likely to develop in P-gp overexpression tumor cells than that in naturally low P-gp expression ones. Huang^[29]^ clarified that the drug released by nanoparticles into the cytoplasm is susceptible to P-gp-mediated drug efflux. Based on the above facts, P-gp-induced drug efflux is considered one of the major reasons for drug resistance.

### Tumor microenvironment and the enzyme system

The tumor microenvironment (TME) refers to the environment in the tumor cells, including mesenchymal cells and capillaries, the secretion of extracellular matrix, factors, and so on. TME contains a variety of stromal cells, which are mainly composed of tumor-associated macrophages (TAMs). As a heterogeneous population with plasticity, macrophages in TAMs exhibit two polarization states during maturation and differentiation through two different activation pathways, namely classical activation type (M1) and alternative activation type (M2), among which M2 TAMs are believed to play a key role in tumor resistance and disease progression^[30]^. In short, TAMs can participate in the generation of drug resistance in tumor cells by releasing cytokines, regulating signaling pathways, intervening in angiogenesis, and interacting with tumor stem cells^[31]^.

Compared with normal tissue, the tumor microenvironment has abnormal physiological and biochemical characteristics, such as low extracellular pH, hypoxia, high intracellular glutathione (GSH) levels, and others. It affects the occurrence and development of tumors, including proliferation, angiogenesis, invasion, migration, drug resistance, and so forth^[32-36]^. It is reported that Hypoxia-inducible factor-1α (HIF-1α) can significantly increase the expression of ABC transporters (MDR1, MRP1, BCRP) to induce cell resistance^[37,38]^. In addition, HIF-1α is found overexpressed in breast cancer and enhances the drug resistance of MCF-7 cells to tamoxifen by mediating tumor cell autophagy^[39]^. GSH is a tripeptide, and glutathione transferase can catalyze glutathione. GSH plays a key role in protecting cells from oxidative stress. Importantly, it not only regulates the pH of the microenvironment in drug-resistant tumor cells, but also plays a key role in regulating cell resistance to drugs. GSH can indirectly enhance drug resistance by inhibiting the RAS-MAPK pathway and activating protein kinase^[40,41]^. Topoisomerase II can form the Topo DNA complex with drugs, and thus inhibit the expression of Topo or increase the phosphorylation level, reducing the enzyme content and activity, and leading to the reduction of drug action targets and drug resistance^[42]^. The Ca^2+^-dependent protein kinase system is also one of the enzyme systems that promote drug resistance in tumor cells. MAPK pathway is one of the important downstream targets regulated by calmodulin-dependent protein kinase II (CaMKII). CaMKII can regulate the survival and proliferation of MDR cancer cells by director indirectly up-regulating protein kinase C (PKC), extracellular signal-related kinases 1 and 2 (ERK1/2), AKT1, and other signaling pathways. PKC is widely distributed in various tissues and cells, which can mediate signal transduction and regulate gene expression and is related to tumorigenesis and drug resistance^[43-45]^.

In recent studies on some biomarkers overexpressed in the tumor microenvironment, many targeted nanoparticles have been developed for drug delivery. Excitingly, these studies also found that some nanoparticles can competitively bind to drug-resistant cytokines and enzymes, thereby blocking the proliferation of drug-resistant cells.

### Transdifferentiation and tumor cell resistance

Under the effect of anticancer drugs, the tumor cell adopts distinct phenotypes to adapt to changes in their environment - This representation is commonly referred to as cell plasticity^[46]^. This characteristic of transforming cells into tolerant or resistant cell states/phenotypes no longer relies on existing drug targets and pathways, thus escaping drug attacks^[47]^. The earliest clinical evidence of cell plasticity came from patients with rare non-small cell lung cancer (NSCLC) who showed resistance to epidermal growth factor receptor (EGFR) inhibitors, and some of the patient’s tumor tissue type transformed into small cell lung cancer (SCLC). Interestingly, Some patients regained sensitivity to EGFR inhibitors after discontinuation^[48]^. Notably, there were differences in cell phenotypes after the transdifferentiation of resistant cells. In another case of a patient with non-small cell lung cancer, the drug-resistant cells metastasized into neuroendocrine cells^[49]^. The most common phenomenon of drug-induced tumor cell plasticity is epithelial-mesenchymal cell transformation (EMT)^[50]^. In addition, lineage transdifferentiation and proliferation-invasion phenotypic transformation are also manifestations of cell plasticity. EMT is regulated by transcription factors mainly from SNAIL, TWIST, and ZEB families^[51-53]^. TGF-β, FGF, EGF, HGF, IGF-1, and members of the Hedgehog, Notch and Wnt signaling pathway - can induce EMT^[54-56]^.

### Regulation of cell metabolism in drug-resistant tumor cells

A common feature of tumor cell resistance is as much as possible to reduce glucose consumption by mitochondrial respiration. In many experiments, biological processes related to mitochondria were observed to be activated or enhanced in tumor-resistant cells. In addition, different types of tumors may differ in how they metabolize glucose. Superoxide produced by mitochondrial aerobic respiration can cause oxidative stress in tumor cells^[57-59]^. Therefore, tumor-resistant cells require a potent antioxidant process in response to superoxide^[60,61]^. One of its antioxidant pathways is that glutathione peroxidase 4 (GPX4) catalyzes the reduction of glutathione. GPX4 is used to reduce intracellular lipid peroxide to reduce oxidative stress^[44]^. The other important antioxidant pathway is aldehyde dehydrogenase (ALDH) which protects drug-resistant cells from the toxic effects of reactive oxygen species^[62]^.

Cells spend more than half of their energy on protein synthesis, so tumor-resistant cells must minimize their protein synthesis^[63]^. Whole-genome studies of different tumors have shown that the proliferation slowdown is deeply related to reduced protein synthesis. Posttranscriptional modification of N6-methyladenine (N6-methyladenosine, m6A) has been found to reduce the translation efficiency of drug-resistant tumor cells in leukemia and melanoma^[64]^.

### Regulation of apoptosis in drug-resistant tumor cells

The eventual aim of most anticancer drugs is to promote tumor cell death. Consequently, the destruction of the apoptosis mechanism may give rise to the drug resistance of anticancer drugs. Defects in apoptosis occur when genes that control apoptosis are activated and are thought to complement the activation of proto-oncogenes^[65]^. Thus, many unregulated oncoproteins, such as MYC, B-cell leukemia protein 2 (Bcl-2), and p53, have a weakened role in triggering apoptosis, which results in the drug resistance of cancer cells^[66]^.

In the apoptotic pathway, the changes of influential factors such as Bcl-2, nuclear factor NF-kappaB (NF-κB), and tumor necrosis factor (TNF) reduce the sensitivity of tumor cells to drugs, and thus inhibit the apoptosis of tumor cells and lead to drug resistance in tumors^[67,68]^. Among them, the molecular mechanism of the Bcl family inducing apoptosis is one of the few apoptosis pathways targeted by Food and Drug Administration (FDA) approved drugs^[69]^. Recent studies have identified another anti-apoptotic protein, myeloid cell leukemia sequence 1 (MCL-1), as a compelling reason for adaptive drug resistance in tumor cells treated with a series of targeted therapies (including BRAF inhibition and EGFR inhibition)^[67]^. Other anti-apoptotic mechanisms may also comprise the trigger of exogenous apoptotic pathways or autophagy^[70-72]^.

### Multidrug resistance and autophagy

Autophagy is a highly conserved biological phenomenon, widely existing in eukaryotic cells^[73]^. It is a way for cells to maintain homeostasis of the intracellular environment and genome stability by self-decomposing damaged organelles and cell components under extreme stress conditions, which is conducive to making cells gain survival advantages under stress and pressure conditions caused by growth or environmental changes^[74]^. An increasing number of studies indicate that certain drugs can induce autophagy in tumor cells when they are stimulated. On the one hand, autophagy, as the executor of type II programmed death, can initiate the autophagy death mechanism and lead to cell death. On the other hand, autophagy, by protecting the survival of tumor cells, may activate the tolerance of tumor cells to chemotherapy drugs, resulting in reduced sensitivity of tumor cells to chemotherapy drugs and drug resistance^[75]^. The up-regulation of autophagy function is a significant factor in drug resistance in drug therapy and radiotherapy. It has been found that the P13K-AKT-mTOR signaling pathway is one of the main pathways mediating protective autophagy, which is mainly involved in the signal transduction of growth factors and hormones^[76]^. In addition, Autophagy related genes (ATGs), p62, and IL-6 can also mediate autophagy to make some cancer cells acquire drug resistance^[77-79]^.

### Enhanced DNA damage repair causes drug resistance in tumor cells

Another possibility for resistance to various anticancer drugs is the enhanced ability of tumor cells to repair DNA damage. Clinical use of cisplatin, DOX, and other chemotherapy drugs can cause DNA damage, which has a significant therapeutic effect. However, repeated DNA damage can induce the abnormal activity of the DNA repair system, leading to increased synthesis of enzyme proteins in tumor cells, weakening the effect of tumor drugs, and resulting in drug resistance^[80]^. When the cell receives the stimulation after injury, it will activate the DNA damage response (DDR) way to reply. Currently, there are at least five major DNA repair pathways - base excision repair (BER), nucleotide excision repair (NER), mismatch repair (MMR), homologous recombination (HR), and non-homologous end joining (NHEJ). They are active at all stages of the cell cycle and enable cells to repair DNA damage^[81,82]^. It is reported that DNA repair endonuclease xeroderma pigmentosum group F (XPF) and DNA repair protein excision repair cross-complementing 1 (ERCC1), which are involved in the nucleotide excision repair (NER) pathway, are crucial to effectively repair DNA damage induced by cross-linking agents and platinum-based reagents. Studies have shown that the overexpression of XPF and ERCC1 proteins is significantly related to the enhancement of cisplatin resistance of cancer cells^[83]^. In many tumors, it was also found that the reduction of the DNA mismatch repair pathway can also lead to the increase of drug resistance, and the hypermethylation of the human MutL Homolog 1 (*hMLH1*) gene promoter leads to the decrease of MLH1 protein expression involved in the mismatch repair pathway. 5-fluoro-2-deoxycytidine and decitabine can reverse this hypermethylation and increase the sensitivity of cells to cisplatin^[84]^.

### Drug target changes in drug-resistant tumor cells

Drug-resistant tumor cells can also acquire drug resistance through gene mutation in the condition of slow proliferation. Therein, mutations or aberrations of targets are one of the most common mechanisms. From the Cancer Genome Atlas (TCGA) database, people can accurately identify mutation types in thousands of tumors using sequencing methods^[85]^. Among them, the activating mutations of oncogenes such as EGFR, RAS, RAF, and PI3K are the key driving factors for many cancers^[86-89]^. With more in-depth research on this mutation generation, many approved drug molecules have been discovered. Many genetic changes lead to the inactivation of tumor suppressor genes in tumor cells, such as PTEN and p16INK4a^[90,91]^. In addition, these mutated proteins may remain permanently “on” or more activated than wild-type (WT) proteins. These activated protein targets, further mutations of amino acids can also lead to drug resistance through numerous mechanisms. Although the mechanism of drug resistance is a highly concerning clinical problem, if we can grasp the off-target mechanism, it may provide a new choice for follow-up targeted drug therapy.

In conclusion, the important molecular mechanisms related to tumor resistance have been extensively investigated, including the ABC transporter superfamily, enzyme system in vivo, cell apoptosis, autophagy, embryonic stem cells, etc., which can lead to increased drug efflux, enhanced detoxification mechanism, increased DNA repair capacity, elevated metabolism of xenobiotics, and cell death inhibition. Due to the interaction of genes and signal regulatory pathways, these mechanisms can not clearly explain the MDR mechanism of tumors in a certain way. With the further advancement of the research on transcriptome and proteome, the regulatory mechanism of MDR can be explained in a multi-dimensional way, providing a reference for the research on the clinical reversal of tumor resistance.

**Figure 2:**
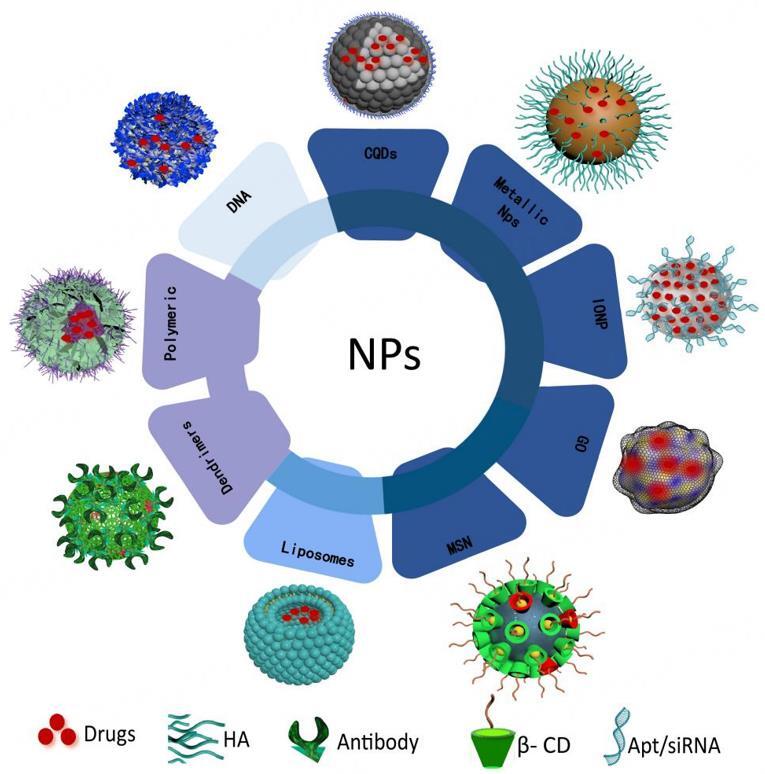
The application of nano-loaded drug system in anti-drug resistance of tumor cells. The advantage of EPR effects could increase the aggregation of drugs in cells, surface-modified antibodies, aptamers, and folic acid can increase the targeting effects, and drugs can also be encased in nanoparticles to escape the recognition of efflux proteins. CQDs: Carbon quantum dots; EPR: enhanced penetration and retention; GO: graphene oxide; HA: hyaluronic acid; IONP: iron oxide nanoparticle; MSN: mesoporous silica nanoparticle; NPs: nanoparticles; β-CD: β-cyclodextrin.

Drug resistance of tumor cells can occur in any part of tumor cell growth. With the rapid development of modern medical technology, scientists continue to uncover the mystery of drug resistance in tumor cells. Subsequently, a new generation of antitumor drugs with targeting and high efficiency has been developed continuously. However, these chemotherapy drugs still have the shortcomings of cross-resistance, off-target, and damage to normal tissues. Due to the advantages of small particle size, large specific surface area, safety, enhanced permeability and retention effect (EPR effect), and promotion of drug enrichment, nanocrystals have been widely used^[92]^. For the past few years, it has become a hot topic for scientists to effectively improve the therapeutic effect of antitumor drugs by using nanocarriers to load drugs and external tumor-specific identification of target molecules to establish targeted nano-delivery systems [Figure 2 and Table 1]. In the following, we will introduce some principles of drug resistance in tumor cells, as well as nano-drug delivery strategies targeting these resistance mechanisms.

**Table 1:** Characteristics based on partial nano drug delivery systems

**Classification**	**Nanocarriers**	**Nanocarriers properties**	**Disadvantage**
Organic nanocarriers	Liposomes	(a) Amphiphilic, biocompatible(b) Wide adaptability(c) Targeting potential(d) Ease of modification	(a) Poor stability, easy to be affected by metal radiation, high temperature, pH, and enzymes(b) Low drug loading rate
	Polymeric micelles	(a) Long retention time in the body(b) Suitable carrier for water-insoluble drug(c) Ease of functional modification(d) Biocompatible, self-assembling, biodegradable(e) Special “core-shell” structure, targeting potential	Poor physical stability, resulting in drug leakage and sudden release
	Polymeric nanoparticles	(a) Water-soluble, nontoxic, and biodegradability(b) High drug loading(c) Selective accumulation and retention in tumor tissue (EPR effect)(d) Active targeting and smart response	(a) Easy binding to negatively charged non-specific cells or proteins(b) Low gene transfection efficiency
	DNA/RNA	(a) Easy synthesis and modification(b) Low immunogenicity(c) Excellent specificity and affinity(d) Active targeting and Intelligent drug release	(a) Poor cellular uptake(b) Poor stability
	HSA	(a) Safety, no immunogenicity, good biocompatibility(b) Biodegradable(c) Passive targeting	(a) Large particle size and easy degradation(b) Preparation method is easy to cause increased toxicity(c) Limited sources of HSA
Inorganic nanocarriers	Metal nanoparticles	(a) Biocompatible(b) Easy preparation	(a) Need surface modification(b) Poor biocompatibility
	Non-metallic nanoparticles		(a) Low surface potential(b) Low drug loading(c) Easy aggregation

EPR: Enhanced permeability and retention; HSA: human serum albumin.

Nanoparticles, as exogenous substances, will encounter various obstacles at all stages of transportation in vivo. After being injected intravenously into the blood, various proteins in the plasma will be attached to the surface of the nanoparticles and labeled. The reticuloendothelial system and mononuclear macrophages will recognize and remove the labeled nanoparticles and even form vascular embolism or organ infarction. By changing the characteristics of nanoparticles, such as stability, surface charge, surface functional groups, etc., we can improve the circulating ability of nanoparticles in vivo, increase the aggregation and targeting of drugs, and finally reverse the drug resistance of tumors.

## NANO DRUG DELIVERY STRATEGY FOR MDR TREATMENT

The nanoparticle delivery platform can regulate drug release and change drug distribution in vivo. Currently, commonly used nanoparticle carriers include liposomes, solid lipids, polymers, mesoporous silica, and inorganic nanoparticles^[93,94]^. It has been shown that nanoparticles can be designed with different sizes, multiple surface modifications, different shapes, and multiple loading modes^[95]^. It can improve the solubility and bioavailability of insoluble drugs and enhance the targeting of drugs. At the same time, light, heat, sound, and other stimuli to trigger the release of drugs can reduce the toxic side effects of drugs^[96,97]^. In the following, we detail the evolution of nanoparticles for overcoming chemoresistant tumors.

The tumors in the clinic are divided into solid tumors and liquid tumors. Solid tumors refer to a class of detectable tangible masses with high clinical incidence^[98]^. However, non-solid tumors are mainly tumors of the hematopoietic system and lymphatic system, accounting for only 10% of all tumors, and can not be seen in imaging examinations^[99]^. Unlike organ cancer and other solid tumors, blood cancer (including multiple myeloma, leukemia, and lymphoma) is formed in the bone marrow or the lymphatic system. Existing treatments for blood cancers include chemotherapy, radiation, immunotherapy, and transplantation^[100,101]^. Chemotherapy is the primary treatment for liquid tumors. However, as anticancer drugs are easy to produce drug resistance, the survival rate of patients is low, so the recurrence rate is high. Therefore, there is an urgent need for a targeted drug delivery system to improve the effectiveness of solid and liquid tumor therapy. After modification and modification of nanomaterials, nanocarriers can be given the ability to actively target and passively target tumor cells (including microenvironment, receptors, ion channels, organelles, etc.), which can increase the chemical drugs enrichment in drug-resistant tumor cells and safety. Meanwhile, nanocarriers themselves or cooperatively delivered various drugs can resist tumor drug resistance through phototherapy-chemotherapy, photothermal-chemotherapy, immunochemotherapy, gene therapy-chemotherapy, and other ways. Different types of nanoparticles have received considerable attention in the therapy of various solid tumors, leading to several successful drug delivery systems entering clinical practice over the years [Table 2]. In the following, we detail the evolution of nanoparticles for overcoming chemoresistant tumors. Table 2 provides a brief selection of currently approved and investigational nanomaterials in the arena of chemoresistant anticancer therapeutics (approved references are 102 to 109). Table 3 presents the example mentioned in subsequent sections.

**Table 2:** Representative examples of nanocarrier - marketed products or products in clinical trials with their indications

**Classification**	**Compound**	**Indications**
Liposome	Liposomal doxorubicin (Doxil, Janssen)	Karposi’s sarcoma, ovarian cancer, multiple myeloma
	Liposomal vincristine (Marqibo, Spectrum pharmaceuticals)	Acute lymphoblastic leukemia
	Liposomal irinotecan (Onivyde, Ipsen biopharmaceuticals)	Pancreatic cancer
	Liposomal daunorubicin and cytarabine (Vyxeos, Jazz pharmaceuticals)	Acute myeloid leukemia with myelodysplasiarelated changes
	Liposomal lurtotecan (OSI-211, phase-II)^[102]^	Recurrent ovarian cancer, recurrent small-cell lung cancer
	Liposomal paclitaxel (LEP ETU, phase-I/II)^[103]^	Advanced solid tumors
	Liposomal oxaliplatin (Aroplatin, phase-II)^[104]^	Advanced colorectal cancer
	Liposomal interleukin-2 (Oncolipin, phase-II)^[105]^	Immune stimulant for use with a liposomal vaccine against non-small cell lung cancer
Polymeric	Leuprolide acetate and polymer (Eligard, Tolmar)	Prostate cancer
	Pegfilgrastim (Neulasta, Amgen)	Chemotherapy-induced neutropenia
	Dantrolene sodium (Ryanodex, Eagle pharmaceuticals)	Malignant hypothermia
	Pegaspargase (Oncaspar, Baxalta U.S.)	Acute myeloid leukemia
	PEG-PLA/paclitaxel (Genexol-PM)	Breast and lung cancer
	PLGA/goserelin acetate (Zoladex)	Prostate and breast cancer
	PLGA/leuprolide acetate (Lupron depot)	Prostate cancer and endometriosis
	PLGA/triptorelin pamoatea (Trelstar)	Advanced prostate cancer
	PLGA/leuprolide acetate (Eligard)	Advanced prostate cancer
	HPMA-copolymer-doxorubicin (PK1; FEC28069, phase-II)^[106]^	Lung cancer, breast cancer, and various other cancers
	PEG-camptothecin (Prothecan, phase-II)^[107]^	Various cancers
	Paclitaxel-poliglumex (CT-2103; Xyotax, phase-III)^[108]^	Non-small cell lung cancer, ovarian cancer
Protein nanoparticles	Denileukin diftitox (Ontak, Eisai)	Cutaneous T-cell lymphoma
	Albumin-bound paclitaxel (Abraxane, Celgene)	Breast cancer, non-small-cell lung cancer, pancreatic cancer
Metal-based nanoparticles	Colloidal gold nanoparticles coupled to TNF and PEG-thiol (CYT-6091; Cyt-immune Sciences, phase 1)^[109]^	Solid tumors

PEG: Poly ethylene glycol; PLA: poly lactic acid; PLGA: poly (lactide-co-glycolide); TNF: tumor necrosis factor.

**Table 3:** Examples of nanocarriers as drug delivery vehicles for cancer treatment

**Category**	**Characteristic**	**Composition**	**Drug**	**Cell lines**	**References**
Liposomes	Passive targeting	TPGS-liposomes	Docetaxel	A549/ADR cells	[84]
	Passive targeting	Pluronic P105-liposomes-paclitaxel (PPL)	Paclitaxel, ambroxol	A549/ADR cells	[85]
	Active targeting-CD44 receptor	Hyaluronic acid-liposomes	Baicalein, DOX	MCF-7/ADR cells	[114]
	Active targeting-nucleolin	Liposomes-aptamer (AS1411)	DOX	MCF-7/ADR cells	[128]
Polymeric nanoparticles and polymeric micelles	Passive targeting	PLGA	Vincristine, Verapamil	MCF-7/ADR cells	[91]
	Active targeting	ICG-IONP&PLGA-CS&CCM (PIO-PCSCM)	DOX, Mcl-1 siRNA	MCF-7/ADR cells	[130]
	Active targeting-transferrin receptor	PLGA-(D-penicillamine-Au-Cu)-transferrin	-	MDA-MB-231 cellsMDA-MB-468 cells	[117]
	Passive targeting	PEG	DOX	MCF-7/ADR cells	[136]
	pH-sensitive	PLGA-TPGS	DOX, Chloroquine	A549/Taxol cells	[149]
Dendrimers	Passive targeting	PAMAM-PEG-DOPE	MDR-1 siRNA, DOX	MCF-7/ADR cells	[98]
	Passive targeting	Pluronic F68 (PF68)-polyamidoamine (PAMAM)	DOX	MCF-7/ADR cells	[100]
	Passive targeting	PAMAM-PEG-DOPE-mAb 2C5	DOX, MDR1 siRNA	MDA-MB-231	[154]
Inorganic nanoparticles	pH-sensitive	Polydopamine (PDA) + MSN + ZIF-8 (PDAMSN@ZIF-8)	DOX, Curcumin	MCF-7/ADR cells	[106]
	Passive targeting	Trimethoxysilylpropyl-ethylenediamine triacetic acid (EDT)-IONPs	DOX	MDCK-MDR1 cells	[107]
		graphene oxide (GO)-molecular beacons (MBs)	DOX	MCF-7/ADR cells	[109]
	pH-sensitive	Au nanorod cluster (AuCR)	DOX, Curcumin	MCF-7/ADR cells	[111]
	Passive targeting Photothermal therapy	Phospholipid-poly(ethylene glycol)-multiwalled carbon nanotubes (Pab-MWCNTs)	P-gp antibodies	3T3-MDR1 cells NCI/ADR-RES cells	[137]
	Active targeting-MUC1	MSN-MUC1	Navitoclax, S63845	MDA-MB-231 cells	[142]
	Active targeting	Cancer cell membrane (CCM)-calcium carbonate (CC)	MiR-451, DOX	BIU-87/ADR cells	[155]
	Active targeting-CD44 receptor photothermal chemotherapy	Molybdenum disulfide-(MoS2)	DOX	MCF-7/ADR cells	[160]
	Passive targetingphotodynamic therapy	Acetylated chondroitin sulfate (AC-CS)	Protoporphyrin IX, DOX, Apatinib	MCF-7/ADR cells	[165]
Protein nanoparticles	Active targeting-folate receptor	folate-human serum albumin (FA-HSANPs)	Taxol, 2-ME	EC109/Taxol cells	[115]
	Long internal circulation time	Bovine serum albumin (BSA)	Docetaxel, Quercetin	MDA-MB-231 cells	[147]
	Active targeting-EGFR	Human serum albumin (HSA)	DOX, MDR1 siRNA, Cetuximab	MCF-7/ADR cells	[156]
DNA/RNA	Active targeting-MUC1	MUC1 aptamer-(P-gp and Bcl-2) antisense oligonucleotides	DOX	MCF-7/ADR cells	[127]
Others	Active targeting-folate receptor	Folate-planetary ball milled (PBM) nanoparticles	Resveratrol, Docetaxel	PC3/ADR cells	[175]
	Passive targetingPhotodynamic therapy	Lipid-coated carbon-silicon hybrid nanoparticles (LSC)	DOX	NCI/RES ADR cells	[161]

ADR: Adriamycin; CD44: cluster of differentiation-44; DOPE: 1,2-dioleoyl-glycero-3-phosphoethanolamine; DOX: doxorubicin; ICG: indocyanine green; IONP: iron oxide nanoparticle; MCF-7: breast cancer cells; MDCK: madin-darby canine kidney; MDR: multidrug resistance; ME: mercaptoethanol; MSN: mesoporous silica nanoparticle; MUC1: mucin 1; NCI/RES ADR cells: a multidrug-resistant ovarian cancer cell line; PEG: polyethylene glycol; PLGA: poly lactic-co-glycolic acid; PLGA-CS: (poly lactic-co-glycolic acid)-chitosan; RES: reticuloendothelial system; TPGS: tocopheryl polyethylene glycol succinate; ZIF: zeolitic imidazolate frameworks.

## INCREASE THE DRUG AGGREGATION IN MDR CELLS-PASSIVE TARGETING

The blood environment is a primary factor affecting drug accumulation in tumor tissue. Studies have shown that the blood environment is the first major obstacle for nano-drug delivery systems^[110]^. The reticuloendothelial system (RES), which consists of body blood protein, opsonin, liver, and spleen, plays a prime role in the metabolism and clearance of nano drug loading system. Tumor blood vessels are hyperactive, and there is a gap between endothelial cells and the lymphatic system. Vascular leakage is associated with EPR effects^[111,112]^. Most types of nanocarriers can enhance permeability and retention (EPR) effects^[113]^. Nanoparticles (NP) can accumulate in tumor tissues and obtain passive targeting by utilizing the unique pathophysiological mechanism of tumor blood vessels to overcome the drug resistance of cancer cells. It was found that the key parameters affecting the effect of NPs EPR were the size and surface of NPs, and particles up to 100 nm in diameter could migrate into tumor tissues^[114]^. Therefore, nanoparticles can continuously deliver drugs to tumor tissue without increasing the dose of chemotherapy drugs. As nanoparticles usually carry multiple chemotherapeutic drugs or bioactive components with multiple anti-MDR mechanisms, the in-depth development of nanomaterials will gain more benefits for MDR tumor treatment. We further introduce different kinds of nano-drug-loaded particles in the following.

### Liposomes

The liposome is one kind of nanocarrier formed by encapsulating drugs in a lipid-like bilayer. Liposomes have excellent biocompatibility due to their similar structure to the plasma membrane of biological cells. Liposome encapsulation of drugs can reduce drug toxicity, improve drug stability and bioavailability, and has gradually been widely applied as a delivery carrier for small molecule drugs and proteins^[115]^. Doxil®, a lipid-based nanoparticle drug preparation approved by FDA, has been used in the clinical treatment of a variety of malignant tumors (metastatic breast cancer, ovarian cancer, multiple myeloma, etc.). Subsequently, Marqibo®, DaunoXome®, Vyxeos®, Onivyde®, and other liposome nano preparation have also been approved by FDA for tumor treatment^[116-119]^. The core of the liposome is a hydrophobic region formed by lipophilic groups, and the inside of the lipid bilayer is a hydrophilic region formed by the phospholipid layer, which can co-carry hydrophilic and hydrophobic drugs. Li *et al.*^[120]^ prepared D-α-tocopherol polyethylene glycol-1000 succinate (TPGS) coated liposome (TPGS-liposome) as a drug delivery platform for docetaxel (TPGS-DTX-liposomes). In this drug-loaded nanosystem, liposomes are easily absorbed by cells through endocytosis to increase the cellular uptake of docetaxel. TPGS can prevent Docetaxel (DTX) from being recognized by the P-gp efflux pump when passing through the cell membrane, thus further increasing the concentration of DTX in A549/ADR cells. TPGS-DTX-liposomes are associated with decreased DTX toxicity, increased safety, reversal of MDR, and improved lung cancer therapy. Wang *et al.*^[121]^ prepared a novel pluronic hybrid paclitaxel-loaded liposomes (PPL). They insert Pluronic P105, a P-gp inhibitor, into the phospholipid bilayer of liposomes and simultaneously load paclitaxel. In drug-resistant A549/Taxol and MCF-7/ADR cells, it can be observed that the PPL + ambroxol regimen has better MDR cell sensitization and killing effect in vitro and in vivo. The Ax can inhibit cell autophagy, while Pluronic P105 can reduce the expression of P-gp. This drug delivery system can increase the retention time of the drug in the lung and resensitize the drug-resistant tumor cells. However, the disadvantage of liposome nano preparation is that its stability is relatively poor, and sometimes the drug leakage occurs after the loading of the drug, resulting in toxicity in vivo^[119]^.

### Polymeric nanoparticles and polymeric micelles

Polymer nanoparticles have good biocompatibility, biodegradability, and high drug-loading efficiency^[122]^. Commonly used polymer materials include artificial synthesis and natural availability. The artificial synthetic materials include polyethyleneimine (PEI), polylysine (PLL), polycaprolactone (PCL), poly lactic-co-glycolic acid (PLGA), and polyamidoamine (PAMAM)^[123-126]^. Chen *et al.*^[127]^ designed PLGA nanoparticles to deliver vincristine (VCR) and verapamil (VRP). The synergetic effect of chemotherapy drug proliferation is significantly enhanced in MCF-7/ADR cells. The same results are observed in the MCF-7/ADR tumor-bearing xenotransplantation model in vivo. Coincidentally, people found that micelles have the characteristics of penetrating and accumulating in tumor tissue; therefore, amphiphilic block copolymers have received extensive attention in the development of micellar delivery systems^[128,129]^. In Guo’s^[130]^ work, he developed an intelligent drug delivery system (DDS) to treat drug-resistant BC. The photothermal and magnetic effects of indocyanine green (ICG)-iron oxide nanoparticles (PIO NPs), DOX, and myeloid cell leukemia-1 siRNA (Mcl-1 siRNA) were encapsulated in PLGA-CS NPs. The above NPs were coated by the cancer cell membrane (CCM) of MCF-7 cells to prepare PIO-DOX-siRNA-PCSCM NPs. CCM-encapsulated intelligent drug delivery system (DDS) showed exceptional targeting. At the same time, pH stimulation and near-infrared irradiation accelerate drug release. The efflux of Dox was reduced by NP-treated MCF-7/ADR cells under a magnetic field and near-infrared laser irradiation. And the in vitro cytotoxicity was increased. Drug encapsulation in micelles can improve drug solubility, prolong blood circulation time, reduce side effects, and enhance antitumor activity. So far, many micelles have entered clinical trials or obtained clinical approval. For example, Poloxam, a Planck block copolymer, has been widely used to deliver small-molecule hydrophobic drugs, has been widely used to deliver small-molecule hydrophobic drugs. Genexol-PM polymer micellar nano preparations have been applied for the market and the FDA has also approved the clinical trial^[131,132]^.

### Dendrimers

Dendritic polymer is a type of polymer nanocarrier with a star or branch structure that allows therapeutic or diagnostic agents to couple on its surface to maximize the role of tumor diagnosis and treatment^[133,134]^. Pan *et al.*^[135]^ coupled the fourth-generation dendrimer amino polyamide amine (G4 PAMAM) with a polyethylene glycol (PEG) modified 1,2-dioleoyl-sn-glycero-3-phosphoethanolamine (PEG-DOPE) block copolymer. Amphiphilic block copolymers can be self-assembled into nano micelles, which can be co-loaded with siRNA onto PAMAM groups and encapsulate water-soluble chemotherapy drugs in hydrophobic cores. This dendrimer micelle mixing system co-delivers MDR-1 siRNA and drugs to tumor tissues through the EPR effect. The positive charge of PAMAM can promote siRNA complexation and tumor cell uptake. The DOPE residues and tertiary amines in PAMAM can improve the escape of endosomes and the intracellular transport of active components^[136]^. Wang *et al.*^[137]^ designed a series of novel amphiphilic dendrimer micelle (PAMAM-n-PF68), and the core of the new dendrimer has large voids and a higher drug loading efficiency. The drug-loaded NPs can effectively inhibit drug-resistant cells by increasing drug uptake and reducing drug efflux. In vivo studies show that the dendrimer can not only increase the survival rate of nude mice bearing MCF-7/ADR tumors but also reduce the cardiotoxicity of DOX.

### Inorganic nanoparticles

Nanocarriers of inorganic materials have been widely developed and studied by scholars due to their unique physical and chemical properties (such as optical, electrical, and magnetic properties, etc.), diversity of structure and function, outstanding biocompatibility and excellent biological distribution, and their ability to overcome bio-barriers at the cellular and tissue levels^[138,139]^. Among inorganic nanoparticles, magnetic nanoparticles, mesoporous silica nanoparticles, carbon nanoparticles, quantum dots and nano-gold have been widely recognized and concerned by scholars as antitumor drug carriers due to their respective characteristics and advantages^[140-142]^. Wang *et al.*^[143]^ prepared a core-shell nanocomposite, polydopamine (PDA) mediated integration of the mesoporous MSN core and the microporous zeolite imidazolate frameworks-8 (ZIF-8) shell (PDAMSN@ZIF-8), ZIF-8 and curcumin (CUR, a P-gp inhibitor) were used for plugging to prevent the premature release of DOX from the mesoporous core. Under acidic conditions, the drug-loaded nanoparticles can release CUR and DOX successively, and the CUR is preferentially released into the cytoplasm to inhibit the efflux of P-gp. These NPs realize the high capacity load of DOX and with continuous drug release characteristics.

Iron oxide nanoparticles (IONPs) are one of the most common inorganic nanocarriers. They are biocompatible and can be incorporated into the iron cycle after being degraded in vivo. Norouzi *et al.*^[144]^ developed nanoparticles with biocompatible IONPs stabilized with nanoparticles of trimethoxysilylpropyl-ethylenediamine triacetic acid (EDT). The drug delivery system can provide magnetic targeting for specific sites. At the same time, it prolongs the circulation time of DOX in vivo and increases DOX aggregation in MDR glioma cells.

Among the numerous carbon-based nanoparticles, graphene oxide (GO) has attracted much attention due to its favorable biocompatibility and tolerable toxicity as a nanocarrier for drug delivery^[145]^. In Li’s research^[146]^, a new drug-loaded nanosystem consisting of graphene oxide (GO) modified with two molecular beacons (MBs) and DOX was developed. When the nanosystem was uptaken, DOX was released into acidic endosomes, and MBs modified on the surface of GO can silence intracellular multidrug resistance 1 (MDR1) mRNA and upstream erythroblastosis virus E26 oncogene homolog 1 (ETS1) mRNA, effectively inhibit the expression of P-gp, further prevent DOX efflux.

Gold nanoparticles (AuNPs) are ideal nanocarriers with a small volume ratio, large surface area, high biosafety, easy synthesis, and strong reactivity to cells. Even more remarkable, the gold nanoparticles showed photothermal effects when irradiated by a near-infrared laser at 808 nm. Compared with a single gold nanoparticle, the photothermal or photoacoustic imaging effect of its clusters or combinations is more prominent, with improved drug loading ability, enhanced tumor targeting, and increased drug accumulation in tumors^[147]^. Wang^[148]^ prepared an Au nanorod Cluster (AuCR) using the emulsion/solvent evaporation method by self-assembly of DOX and amphibious poly (curcumin-co-dithiodipropionic acid) b-biotinylated poly(ethylene glycol) with Au nanorod (AuNR). The AuCR can be decomposed into an independent AuNR unit at an intracellular concentration of 5 mM GSH to trigger DOX release at pH (pH 6.8 or 5.0). The system can increase DOX uptake in ADR tumor cells and inhibit DOX exportation.

## NPS TARGETING THE DRUG-RESISTANT TUMOR CELL BIOMARKERS-ACTIVE TARGETING

Active targeting is a high-performance method to deliver the “cargo” (such as therapeutic drugs and genes) into drug-resistant tumor cells by specific targets without affecting healthy tissues, with better therapeutic effects and less toxicity^[149]^. The outermost layer of drug-loaded nanoplatforms with active targeting functions mainly consists of different types of targeted ligands, such as antibodies, peptides, aptamers, and small molecules. Generally, folate, hyaluronic acid, cell-penetrating peptide, transferrin, and biotin are common targeting modification molecules^[150]^. For instance, Liu *et al.*^[151]^ prepared hyaluronic acid-modified liposomes containing baicalein and DOX. In vivo and in vitro studies show that compared with DOX alone, the prepared target functional nanoparticles have a stronger inhibitory effect on the proliferation of breast cancer-resistant MCF-7/ADR cells. Liu *et al.*^[152]^ prepared folate-bound human serum albumin nanoparticles (FA-HSANPs) that carry taxol and the chemical sensitizer 2-methoxyestradiol (2-ME). Due to the combination of folic acid and folic acid receptors, the drug-loaded nanoparticles entering EC109/Taxol cells are increasing. The co-encapsulation of PTX and 2-ME in FA-HSANPs can prolong the circulation time, improve the antitumor effect, and reduce the side effects of PTX. It also exhibits highly potent cytotoxicity and apoptosis-inducing activities in the G2/M phase of PTX-resistant EC109/Taxol cells. Meanwhile, 2-ME can effectively reduce the expression of drug resistance-related proteins in EC109/Taxol cells and reverse its MDR to PTX to some extent.

Epithelial-mesenchymal transformation (EMT) in solid tumors promotes tumor progression, MDR, migration, and differentiation^[153]^. Shome *et al.*^[154]^ used Poly (D,L-lactic-co-glycolic acid) (PLGA) as the core, the secondary layer covered with Au-Cu nanoclusters, and the outermost layer are transferrin. It can target the transferrin receptor overexpressing triple-negative breast cancer (TNBC) and have available cell internalization ability as well as highly anti-cell proliferation ability. In addition, in EMT-induced TNBC cells, reactive oxygen species (ROS) are produced after co-incubation with nanocomposites, which can induce apoptosis by altering mitochondrial membrane potential. At the same time, the down-regulation of EMT reduced the migration ability of TNBC cells. A 4.63-fold reduction in ABCC1 expression was observed in MDA-MB-231 cells.

The aptamer is a kind of short-chain oligonucleotide with high sensitivity, biocompatibility, biodegradability, and poor immunogenicity. By modifying aptamer sequences on the nanoparticles, selective active targeting of ligands can be achieved^[155,156]^. Moreover, aptamers have the advantages of convenient synthesis, chemical modification, and high stability. In this regard, some tumor biomarkers that can be detected by targeting ligands include epithelial cell adhesion molecule (EpCAM), mucin-1 (MUC1), nucleolin, prostate-specific membrane antigen (PSMA), HER2, etc.^[157-160]^. Recently, many targeted aptamers have been studied, including AS1411, 5TR1, HApt, and so on^[161-163]^. Pan *et al.*^[164]^ used DNA origami technology to assemble MUC1 aptamer functionalized rectangular origami (Apt-origami) with P-gp and Bcl-2 antisense oligonucleotides (Apt-origami-ASO) and then load Dox (Apt-DOA). This Apt-DOA shows good lysosomal escape and drug-responsive release in Hela/ADR and MCF-7/ADR cells. Simultaneously, it can also significantly silence Bcl-2 and P-gp proteins and induce cell apoptosis.

Nucleolin is a membrane shuttle protein that is overexpressed in the cytoplasm and cell membrane in metastatic and rapidly dividing breast cells. Li *et al.*^[165]^ developed a nucleolin-targeting delivery system [Lip (Ap-Dox)] with AS1411-DOX on the inner layer and liposome on the outer layer to avoid drug resistance in breast cancer (MCF-7/Adr). The advantage of the above NPs is the transmembrane affinity of liposomes to deliver nanoparticles into cells and release Ap-Dox, which increases the accumulation of Ap-Dox in cancer cells. In addition, this targeting of AS1411-nucleolin can accumulate Dox in the nucleus, thus highly enhancing its therapeutic efficacy against drug-resistant cancer cells by effectively bypassing P-gp. This novel nano-drug treatment strategy for breast cancer provides different insights into the future design of NP-delivery drugs.

### NPs target efflux and transfer system to overcome tumor resistance

One way to overcome the MDR controlled by ABC transporter is to use ABC transporter inhibitors to make tumor cells sensitive to chemotherapy drugs. ABC transporter inhibitors were combined with anticancer drugs to improve drug sensitivity. However, the toxicity of the MDR reversal agent limits its application. Nevertheless, the enthusiasm for ABC efflux transporters to overcome MDR is still high. Delivery of drugs into cells by employing a nanocarrier can enhance the accumulation of nano drugs in cells and reverse the efflux of ABC transporters. This method has become one of the most effective methods to overcome the drug resistance of tumor cells^[166]^.

Currently, commonly used MDR effector inhibitors include cyclosporine, verapamil, Taliqueta, and curcumin^[167,168]^. The scientists also found that some surfactants also have anti-MDR effects, such as Solutol®HS15, Cremophor®EL, Tween®80, and TPGS, all of which contains PEG in their hydrophilic parts.^[167,169-171]^. These nanoparticles are also good carriers for drug delivery. Zhang *et al.*^[172]^ studied the effect of the surface charge of PAMAM dendrimer on the exocytosis and mechanism of multidrug-resistant tumor cells. In drug-resistant human breast cancer cells (MCF-7/ADR cells), exocytosis kinetics, pathway, and mechanism were systematically analyzed. The results prove that the positive charge on the surface of PAMAM dendrimers promoted exocytosis, while the neutral and negatively charged dendrimers had a slight effect on exocytosis in MCF-7/ADR cells. PAMAM-NH2 with positive charges is more likely to be distributed in mitochondria and nuclei, which is an ideal mitochondrial targeting agent and gene-drug carrier for multidrug-resistant tumor therapy.

Mao *et al.*^[173]^ designed polyethylene glycol (PEG, MW: 2K) to deliver DOX (PEG_2K_-DOX). PEG_2K_-DOX nanoparticles significantly increased the uptake and cytotoxic activity of MCF-7/ADR and KBv200 to DOX, and their IC50 values decreased to 1.130% and 42.467%, respectively. Compared with doxorubicin, PEG_2K_-DOX nanoparticles shows significantly improved plasma pharmacokinetics, enhanced in vivo therapeutic efficacy against MDR xenografts, and better safety in vivo. Suo *et al.*^[174]^ has studied and synthesized a multiwalled carbon nanotubes dense coating with phospholipid-poly(ethylene glycol) (Pab-MWCNTs). By connecting MWCNTs to P-gp antibodies, the combination of body targeting and laser-guided heat therapy was realized. Pab-MWCNTs can penetrate deeply within tumor spheroids, but after brief exposure to near-infrared, both P-gp-transfected 3T3 cells and P-gp-expressing drug-resistant cancer cells showed higher lethal activity. Singh *et al.*^[175]^ prepared a novel nano-drug system called the planetary ball milled (PBM) nanoparticles (FA-RES + DTX-NP) that were coated with resveratrol (RES), combined with docetaxel (DTX), and surfacing coupled with folic acid (FA), which could enhance the bioavailability of resveratrol (RES) and DTX to treat advanced metastatic PCa. Planetary milling technology has attracted more and more attention for the advantages of preparing nanoparticles without additive or final dewatering steps to recover nanoparticles. Over the years, the ball milling process has been explored for nano reduction of different polymer materials, including starch, cellulose, and protein-based polymers, for different applications^[176,177]^. The cytotoxic effect of FA-RES + DTX-NP nanoparticles is very effective and can reduce the concentration of free drug (DTX) by 28 times. After FA-RES+DTX-NP treatment, the expressions of NF-kB p65, COX-2, pro (BAX, BAK), and anti-apoptotic (Bcl-2, Bcl-XL) genes were significantly decreased. In addition, treatment of DTX-resistant PCa cells with FA-RES + DTX-NP negatively affected the ABC transporter marker, thereby limiting the multidrug-resistant phenotype of cancer cells and effectively enhancing the intracellular concentration of the drug, thereby exerting its cytotoxic effects.

## NANO DRUG DELIVERY STRATEGY FOR REGULATING APOPTOSIS AND METABOLISM-RELATED FACTORS

In the human body, apoptosis is a highly programmed cell death process^[178]^. When apoptosis is defective, it may also lead to drug resistance to chemotherapy drugs. And this defect may be caused by the inactivation of apoptosis-promoting genes and activation of anti-apoptotic proteins of the Bcl-2 protein family, as well as survival signal proteins (Survivin, FLIP, and NF- κB)^[65]^. In the development of fighting against drug resistance of tumor cells, people have studied the proteins related to the regulation of apoptosis and developed inhibitors and drug-loaded nanoparticles that can interfere with the process of apoptosis. Recently, inhibitors of Bcl-2 and MCL-1 anti-apoptotic proteins have been developed to overcome drug resistance, such as ABT-263 (Navitoclax) against Bcl-2 and S63845 against MCL-1^[179-181]^. For example, Vivo‐Llorca *et al.*^[182]^ prepared MSN-apMUC1 containing Navitoclax and S63845. By modifying the transmembrane protein MUC1 aptamer overexpressed in breast cancer cells on the surface of mesoporous silica nanoparticle (MSN), the system can be actively targeted to the MDR cells. In addition, the MSN gap also carries an inhibitor of anti-apoptotic proteins. The drug-loaded nano platform could effectively target Triple-Negative Breast Cancer (TNBC) cells and successfully induce apoptosis. The drug delivery system improves the antitumor activity and is effectively targeted to TNBC cells.

The down-regulation of the *C-Myc* gene contributes to the occurrence of a series of human tumors by regulating endogenous and exogenous apoptosis pathways^[183]^. In Wang’s^[184]^ study, it was confirmed that multiwalled carbon nanotubes (MWCNTs) could reduce the expression of ABC transporter in human colorectal adenocarcinoma Caco-2 cells by regulating the expression of C-Myc. Overexpression of C-Myc reversed the inhibitory effect of MWCNT on ABCB1 and ABCC4 expression.

## COMBINATION-BASED NANOPARTICLE APPROACHES DIRECTED AGAINST MDR

### Nano-drug platform co-delivers chemotherapeutic drugs and sensitizers

In addition to the emerging P-gp inhibitory nanomaterials mentioned earlier, some products extracted from natural plants have also been gradually proven to have anti-P-gp efflux effects. These natural products mainly include alkaloids, coumarins, flavonoids, and terpenes^[185]^. For example, quercetin (QT) in flavonoids has been shown to induce apoptosis, inhibit angiogenesis, and inhibit oxidative stress and mutation in several human cancer cells^[186]^. Desale *et al.*^[187]^ synthesized a novel intravenous bovine serum albumin (BSA) co-loaded Docetaxel (DTX) and quercetin (QT) nanoparticle (DTX-QT-BSA-NPs). In this drug delivery platform, BSA plays a role in prolonging the circulation time of nanoparticles in vivo and the active targeting of NPs to tumors. QT is used as a chemical sensitizer and is first released to inhibit the action of P-gp efflux transporters. The in vivo assay demonstrates that the two drugs significantly enhance the accumulation of DTX in MDA MB-231 (P-gp efflux sensitive) cells by nanoparticle encapsulation compared with free DTX and DTX-BSA-NPs. DTX-QT-BSA-NPs increased the titer of therapeutic drugs in tumor cells.

In recent years, scientists have observed that the anti-malarial drug, chloroquine (CQ), is an inhibitor of lysosomes. CQ can inhibit the fusion of lysosomes and affect the degradation of late autophagy^[188]^. It is a potential natural chemosensitizer. Sun *et al*.^[189]^ prepared Poly(lactic-co-glycolic acid) (PLGA) and D-alpha-tocopheryl polyethylene glycol succinate (TPGS) as carriers to co-delivered DOX and CQ. As a chemical sensitizer, CQ can increase the accumulation of drugs in cancer cells by protecting DOX from autophagy degradation. NP_DOX + CQ_ is pH-sensitive and stable in the neutral environment of normal tissue cells and blood but dissociates in the weakly acidic environment of tumor cells and releases loaded drugs, thus achieving antitumor effects. More importantly, NP_DOX + CQ_ can avoid the recognition of P-gp and thus reduce drug resistance.

### Nano platform co-delivers chemotherapy drugs and genes

Co-delivery of antitumor drugs and genes can improve the effectiveness of treatment. On the one hand, genes can increase the drug sensitivity of tumor cells and thus can reduce the drug’s adverse reactions. On the other hand, it can overcome the problem of low transfection efficiency in gene therapy, which leads to poor therapeutic effects^[190,191]^. Gene therapy usually refers to the therapeutic genes in human target cells in a certain way to correct or replace disease-causing genes and correct gene defects, play the role of biomedical therapy, and achieve the purpose of treating diseases. Small interfering RNA, small RNA, long non-coding RNA, and small hairpin RNA have shown great potential in current-period gene therapy^[192,193]^. They play important roles in regulating tumor of multidrug resistance, the expression of cell cycle-related genes, the expression of DNA repair ability-related genes after cell damage, inducing autophagy, and increasing the sensitivity of chemotherapy drugs to targets. Yalamarty^[194]^ prepared a 2C5-modified MDM with siRNA and DOX (2C5-MDM-D-R). Specifically, the tumor-targeting monoclonal antibody 2C5 (mAb 2C5)-PEG_7k_-DOPE was inserted into a mixed dendritic molecule micelle containing 4th generation (G4) polyamidoamine (PAMAM)-PEG_2k_-DOPE and PEG_5k_-DOPE. The MDR1 siRNA binds electrostatically to the cationic charge on the G4 PAMAM dendrite molecule. The micelle has shown active targeting against tumors in breast (MDA-MB-231) and ovarian (SKOV-3TR) MDR cell lines. MDR1 siRNA inhibited the drug efflux and increased the accumulation of drugs in the tumor cells to reduce the off-target effect. In Wei’s^[195]^ study, a cancer cell membrane (CM) coated with calcium carbonate (CC) nanoparticles was designed to co-deliver miR-451 and DOX to solve the dilemma in bladder cancer treatment (MCC/R-A). The system can target retention and bypass extracellular barriers. In addition, miR-451 overcomes the MDR of cancer cells by down-regulating the expression of P-gp, thereby increasing cellular drug retention in BIU-87/Adr. The therapeutic effect of MCC/R-A on BIU-87/Adr was significantly enhanced, which was better than miR-451 or DOX alone. Yang *et al.*^[196]^ used Human serum albumin (HSA) as the carrier to deliver Dox and MDR1 siRNA simultaneously, and the outer layer was modified with cetuximab. Cetuximab in this nano-loaded system targets overexpressed epidermal growth factor receptors (EGFR) in MCF-7 /ADR cells, so DOX and siMDR1 to tumor sites, enhancing gene silencing and cytotoxic activity.

The synergistic effect of immunotherapy and chemotherapy on different mechanisms is a hot research topic in cancer medicine and has a broader clinical application prospect. Immunotherapy by RNA interference is beneficial to long-term block the interaction of PD-1/PD-L1 (Programmed cell death protein 1 and its ligand) and reduce the proliferation activity of cells^[197,198]^. Tang^[199]^ used mixed micelles to co-deliver Paclitaxel (PTX) and PD-L1 siRNA for melanoma treatment. The mixed micelles could reduce the expression of PD-L1 and p-S6K(mTOR pathway activation markers) in B16F10 cells. Furthermore, cytotoxic T cell immune response is promoted, and mTOR(Mammalian target of rapamycin) pathway activation is inhibited, which can cooperate with chemotherapy to reduce tumor proliferation activity.

### Chemotherapeutic drugs combined with photothermal therapy

Near-infrared laser has the advantages of high efficiency in penetrating tissue and safety, which can be used in tumor treatment. Dong *et al.*^[200]^ designed a molybdenum disulfide (MoS_2_) modified NPs with hyaluronic acid (HA) by taking advantage of its fine particle size and high photothermal conversion efficiency. Under the irradiation of an 808 nm laser, the temperature of MoS_2_ can rise from room temperature to 52 °C within 10 min, which can effectively inhibit the proliferation of drug-resistant tumor cells and significantly reduce the expression of membrane transporters. In vivo experimental observation shows that the tumor growth inhibition rate in the MoS_2_-HA + laser group is as high as 96%, indicating that MoS_2_-HA/DOX can primely deliver drugs and maintain the in vivo drug concentration, improving the effect of combined photothermal chemotherapy. Wang *et al.*^[201]^ successfully constructed a nano drug delivery system with lipid-coated carbon-silicon hybrid nanoparticles (LSC) as the carrier to carry the chemotherapy drug DOX. Under 780 nm laser irradiation, the nanosystem can target mitochondria and produce reactive oxygen (ROS). The ROS can oxidize NADH into NAD^+^ to reduce the amount of ATP available to the efflux pump and promotes MDR cancer cells to return to “normal” for at least 5 days. In vivo data shows that drug-loaded LSC nanoparticles combined with NIR laser therapy can effectively inhibit the growth of multidrug-resistant tumors without significant systemic toxicity. The role of the nanoparticles is also verified in two drug-resistant cell models, paclitaxel-resistant and irinotecan resistant, indicating that this design can effectively overcome the broad-spectrum drug resistance of tumors.

### Chemotherapeutic combined with photodynamic therapy

Photodynamic therapy (photodynamic therapy, PDT) is one of the important adjuvant treatments for cancer. Compared with traditional treatment, PDT is less invasive and less damaging to normal tissues. In addition, it can effectively induce immunogenic cell death (ICD) and stimulate immunity, which is an ideal minimally invasive treatment for tumors^[202,203]^. Photosensitizers mainly include the porphyrin family (hematoporphyrin derivatives, benzoporphyrin derivatives, 5-aminolevulinic acid, Texaphyrins), the chlorine family (Purlytin, Temoporfin, Photochlor), and the dye family (Phthalocyanine, phthalocyanine)^[204]^. The photosensitizers have limitations in clinical applications due to the following properties: poor solubility in water, low chemical purity, and poor tissue penetration. The combination of photosensitizers and nanomaterials can increase the efficiency of PDT as well as eliminate its side effects. In addition, nanoparticles can simultaneously carry chemotherapy drugs and photosensitizers into drug-resistant tumor cells to synergistically inhibit the proliferation of drug-resistant tumor cells. Wei *et al.*^[205]^ used acetylated Chondroitin sulfate (AC-CS) as a long-cycling nano craft and combined protoporphyrin IX (PpIX) onto AC-CS (ACP) via an ester bond. DOX and Apatinib (Apa) can be simultaneously encapsulated into amphiphilic ACP micelles (ACP Dox + Apa). When exposed to 635 nm light, PpIX was activated to produce ROS, then the release of DOX and Apa were triggered. At the same time, excessive ROS have a strong PDT effect on mitochondria or nuclei and eventually lead to apoptosis. This novel nanosystem reverses tumor MDR through Apa-enhanced DOX sensitivity combined with photosensitizer-mediated PDT.

## CONCLUSION

Through continuous efforts, scientists have made great progress in uncovering the mechanisms of drug resistance in cancer. The occurrence of MDR in tumor cells is mainly due to overexpression of transmembrane proteins, abnormal signal transduction pathways, mutations in drug targets or related enzyme systems, dynamic activation of the DNA repair system, and the developing adaptation of cancer cells to the microenvironment, etc., which leads to reduced drug sensitivity. The mechanism of MDR in tumors is very complex, and it is difficult to reverse the drug resistance with a single preparation. However, how to overcome cancer resistance is still an unsolved problem. As more nanomedicine is developed and optimized, the advantages of nanomedicine will become an attractive strategy for reversing or overcoming cancer resistance.

The rational design of a nano drug delivery system can enhance drug solubility, system stability, targeting, and biocompatibility. In general, through the improvement and modification of nanomaterials, the drugs it carries can be more internalized into cells, increasing the concentration of drugs in drug-resistant tumor cells. At the same time, different drugs can also combine with proteins on the cell membrane to reduce drug efflux. Furthermore, these drug-loaded nanoparticles can interfere with the metabolism and apoptosis of drug-resistant tumor cells, silence drug-resistant genes, and downregulate the synthesis of drug-resistant related proteins. In addition, nano drug delivery systems can realize the therapy multi-target and multi-pathway combined, which is one of the future development directions of tumor therapy. It indicates that nanomaterials show a highly competitive application prospect in drug delivery of drug-resistant tumor cells. In this paper, we systematically summarize different strategies based on nanomedicine to improve the efficacy of chemotherapy in drug-resistant tumor cells, including enhancing the drug enrichment of chemotherapy drugs, improving pharmacokinetics, controllable modifications, and reversing the drug resistance of tumor cells in combination with chemotherapy. Compared with traditional preparations, nano drugs can maintain stability in systemic circulation after injection, protect drugs from damage utilizing plasma protein binding, increase circulation time, and realize efficient accumulation and function of therapeutic components in tumor sites. Therefore, the nanocarrier drug delivery with unique functionality and systemic circulation stability is a novel strategy for overcoming MDR. Among the drug-loaded nanoparticles mentioned in this paper, compared with traditional drug-delivery system carriers, polymer micelles have a higher drug-loading capacity, longer drug circulation time, and lower systemic toxicity. Liposomes are used as delivery carriers for water-soluble drugs(such as proteins or DNA), while insoluble drugs are surrounded by a hydrophobic bilayer. However, due to the instability of the membrane, drug loading is limited. Fortunately, PEG-modified phospholipids prevent the recognition and uptake of liposomes by RES, prolong the retention time of drugs in the blood circulation, and can be enriched in tumor cells in tissues or organs other than the liver and spleen by the EPR effect^[206]^. The safe dose of inorganic nanomaterials is uncertain and can cause oxidative stress in normal tissues, which may affect the function of some vital organs. Secondly, most inorganic nanomaterials enter cells through endocytosis, and their uptake by tumor cells directly affects the therapeutic effect. How to improve the targeting of inorganic nanomaterials to tumor tissue is the focus and difficulty of research. In short, the application of nanomaterials in MDR tumors is still in its infancy. The biological safety of nano drug delivery systems still needs to be further explored. These issues include optimizing nano delivery systems to make them more suitable for human use, avoiding or reducing possible toxic and side effects, and degradation and excretion of nanoparticles. In addition, the methods of reversing tumor drug resistance by the nano-drug delivery system still need to be innovated. The efficiency of nano drug delivery systems in MDR tumors can be improved by combining immunology, photodynamics, acoustic dynamics, and other methods may be a key research strategy for future research.

## DECLARATIONS

### Copyright

© The Author(s) 2023.
